# Prolapsus Ani

**Published:** 1907-02

**Authors:** 


### PROLAPSUS ANI.

Newman describes an operation for the cure of prolapsus ani and internal haemorrhoids. Its purpose is to cauterize the mucous membrane of the rectum into six narrow strips from above downwards, each strip being four inches long by a quarter of an inch broad. This is done through a fenestrated speculum six inches long, and one and a quarter inches in diameter, with six windows 4 in. long and one-sixth of an inch broad. When it is fully inserted, the lower ends of the windows are half an inch above the onus, so that the mococutaneous line is protected from the cautery..

A general anaesthetic is given, and any hemirrhoids or prolapsed rectum fully reduced. The speculum with its obturator is then introduced to its full length. On withdrawing the obturator the congested mucous membrane protrudes through the openings in the speculum. A disc on a wire is then passed in to close the upper end of the speculum and protect the mucous membranes higher up. All the mucous membrane protruding in the speculum is then freely cauterized wih an irotn cautery at a dull red heat. A danger to be avoided is overheating the speculum, and it may be met by playing a stream of water upon its interior at intervals. When the operation is completed the speculum is packed with gauze covered with petrolatum, which is held in place by the obturator, while the speculum is withdrawn. Patients should remain in bed three weeks, when union will be found to be complete. In cases of internal piles the patient suffers no pain, and can be out in a few days. The advantages are as follows: 1. The very sensitive area of the anus not being included in the operation, the patient suffers no shock during the operation, and litle or no pain after it. 2. The cautery is applied while the mucous membrane and the muscular wall are as nearly as possible in their normal relationship. 3. The anus being uninjured the sphincter acts normally after the operation. 4. In no cases has the writer found the slightest indication of sepsis after the operation; no suppression of urine, and comparatively little sickness following,an" when the bowels move for the first time no great pain is suffered. 5. In no case has recurrence of prolapse or internal haemorrhiods been noted.—*Lancet, Dec. 22, '06.*

---

### THE RELATIVE THERAPEUTIC VALUES OF VARIOUS OINTMENT BASES.

---

In the *Journal de Medecine de Bordeaux,* 1906, No. 14, is an excellent survey of the various ointment bases which has recently been made by Dr. W. Dubreuilh with the object of showing the necessity of understanding the properties of the various media used as ointment bases in order to secure exactly the desired result. Lard melts at the temperature of the body and is readily absorbed, but it is unsuitable for mixing with compounds of mercury owing to its tendency to become rancid. Soft paraffin does not become rancid, is not acted on by soap or by alkilies, and is not completely removed from the skin by washing in cold water. It is quite an inert substance, and on that account forms a useful basis for chemically active medicaments. While soft paraffin is as readily fusible as lard it differs from the latter in that it remains on the surface, whereas lard penetrates beneath the stratum squamosum. It is valuable, therefore, as a basis for superficial dressings. Wool-fat is useful for giving tenacity to too fluid ointments; it has great penetrating power, is less prone to become rancid than lard, and possesses ths useful property of absording an equal weight of water. Glycerin of starch, made of the consistence of lard, is a penetrating, viscous basis, not prone to rancidity. It is readily removed from the skin by washing. Cerates, composed of a mixture of wax and oil, with or without water, form useful superficial dressings. When they are applied to the skin the oil is absorbed and the water evaporates, leaving behind a layer of wax. They are useful when it is required to apply a medicament to a limited area without diffusion to neighboring parts, hence they occupy an intermediate position between ointments and plasters. An excellent basis for cerates consists of wool-fat and white wax, of each two parts, and olive oil one part. It is advisable always to employ wool-fat when water-soluble substances—e. g., phenol and mercuric chloride—are prescribed.

Dr. Dubreuilh points out that the substances which are incorporated in ointments are not necessarily therapeutically active, and a careful study of their topical action is worthy of attention. Inert powders—e. g., zinc oxide, starch, megnesium carbonate, talc, and kaolin--are frequently added to ointments in order to increase their consistence and to render them more persistent in action, by absorbing a portion of the fatty basis and so hindering its absorption by the skin. This is the explanation of the addition of 10 parts each of starch and oxide of zinc to 20 parts of soft paraffin in the preparation known as Lassar's paste. When the ointment contains in addition an active medicament—e. g., coal tar or sulphur—the action of the latter is prolonged by mechanical dilution with the inert powder. The inert substances mentioned above are preferred by Dubreuilh in the order named; they should be in a very fine state of subdivision. Bismuth subnitrate is sometimes used for the same purpose, but it is not quite inert. Dr. Dubreuilh considers that many active ingredients of ointments—e. g., the various preparations of mercury--act by liberating a volatile substance as a result of decomposition in contact with the skin. Thus in the case of mercurials it is probable that the vapor of mercury is slowly disengaged, while in the case of chrysarobin it is of interest to note that the action of the drug is exerted around, as well as at, the point of application.

From these considerations it is clear that the choice of the ingredients of ointments is by no means a haphazard proceeding. It is of interest to note the opinion expressed by Dr. Dubreuilh that in the case of volatile ingredients—e. g., mercury and methyl salicylate—the choice of a basis is of little importance, the absorption of the active ingredient being practically the same when an impermeable basis—e. g., soft paraffin—is used as when lard is employed as the basis. This point is of importance, since it permits the employment of soft paraffin in such cases in place of lard, which so often turns rancid and so sets up irritation.—London *Lancet,* Oct. 20, 1906.

